# Ribosomal protein L22-like1 (RPL22L1) mediates sorafenib sensitivity via ERK in hepatocellular carcinoma

**DOI:** 10.1038/s41420-022-01153-8

**Published:** 2022-08-17

**Authors:** Dongmei Zhang, Yunzhen Zhou, Yanan Ma, Ping Jiang, Hongchao Lv, Sijia Liu, Yu Mu, Chong Zhou, Shan Xiao, Guohua Ji, Peng Liu, Ning Zhang, Donglin Sun, Haiming Sun, Nan Wu, Yan Jin

**Affiliations:** 1 Laboratory of Medical Genetics, Harbin Medical University, Harbin, China; 2 Key laboratory of preservation of human genetic resources and disease control in China (Harbin Medical University), Ministry of Education, Harbin, China; 3 Department of Histology and Embryology, Harbin Medical University-Daqing, Daqing, China; 4 College of Bioinformatics Science and Technology, Harbin Medical University, Harbin, China; 5 Department of Gynecological Radiotherapy, Harbin Medical University Cancer Hospital, Harbin, China

**Keywords:** Oncogenes, Tumour biomarkers, Growth factor signalling

## Abstract

Precision medicine in hepatocellular carcinoma (HCC) relies on validated biomarkers that help subgroup patients for targeted treatment. Here, we identified a novel candidate oncogene, ribosomal protein L22-like1 (RPL22L1), which was markedly elevated in HCC, contributed to HCC malignancy and adverse patient survival. Functional studies indicated RPL22L1 overexpression accelerated cell proliferation, migration, invasion and sorafenib resistance. Mechanism studies revealed that RPL22L1 activated ERK to induce atypical epithelial-to-mesenchymal transition (EMT) progress. Importantly, the ERK inhibitor (ERKi) could potentiate sorafenib efficiency in RPL22L1-high HCC cells. In summary, these data uncover RPL22L1 is a potential marker to guide precision therapy for utilizing ERKi to enhance the sorafenib efficacy in RPL22L1-high HCC patients.

## Introduction

Hepatocellular carcinoma (HCC) is the second leading cause of cancer death worldwide [1–3]. The prognosis of HCC patients remains dismal due to high incidence of postsurgical metastasis and drug resistance [4, 5]. Despite sorafenib, a multi-tyrosine kinase inhibitor, currently used as the standard treatment of advanced HCC, the survival benefit is limited due to inevitably resistance [6]. Great heterogeneity of HCC is the principal cause of drug resistance and therapy failures. Classifying patients into different fine subgroups by oncogene classification is helpful for effective individualized and accurate treatment. As such, the main problem in clinical diagnosis and treatment of HCC is still the paucity of novel effective oncogenes and biomarkers.

Recently, several homologous analogues of ribosomal protein have been recognized in mammals and were reported to participate in the malignancy of cancer [7–11]. Ribosomal protein L22-like1 (RPL22L1) was a homologous analogue of ribosomal protein L22 (RPL22), and our team previously reported it promoted ovarian cancer metastasis as a novel oncogene [12]. Several more reports indicated that RPL22L1 played pivotal roles in a variety of human cancers, including colorectal cancer [13], prostate cancer [14, 15], and other malignancies [16**–**18]. It was found to regulate diverse cellular functions, including cell proliferation, migration, invasion, apoptosis, DNA repair and drug resistance [12**–**15]. However, the potential role of RPL22L1 in HCC remains still unclear.

Here, we explored the contribution of RPL22L1 in HCC and investigated the therapeutic implications. Our results presented that RPL22L1 overexpression could enhance the malignant phenotype of HCC cells and regulate sorafenib resistance by ERK activation. Pharmacological inhibition of ERK restored the therapeutic effect of sorafenib in RPL22L1-high HCC cells.

## Results

### RPL22L1 expression is frequently elevated in HCC and associates with poor prognosis

**Fig. 1:**
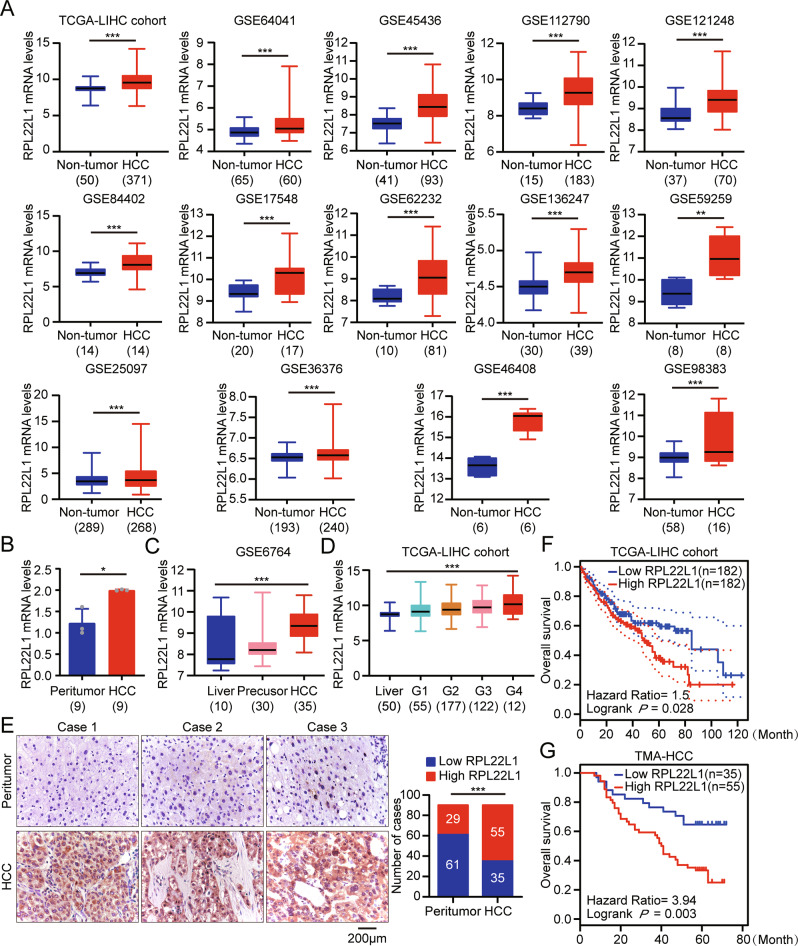
Expression and prognosis importance of RPL22L1 in HCC. **A** RPL22L1 mRNA levels in HCC and non-tumorous tissues were compared through TCGA and 14 independent GEO data sets. **B** RT-PCR analysis of RPL22L1 mRNA in 9 paired clinical HCC and adjacent non-tumor tissues. **C** RPL22L1 mRNA expression during HCC progression from GSE6764 dataset. **D** RPL22L1 mRNA expression in different grades of HCC from TCGA-LIHC dataset. **E** Representative images and score statistics of RPL22L1 IHC staining in 90 paired HCC and adjacent tissues. Case 1–3 represented 3 HCC patients, respectively. Scale bars = 200 μm. Kaplan-Meier survival curves according to RPL22L1 expression in **F** TCGA-LIHC dataset and **G** TMA-HCC data (log-rank test). *P < 0.05, **P < 0.01, ***P < 0.001.

TCGA and GEO databases were used to assess the mRNA expression level of RPL22L1, and found that was statistically higher in HCC tissues than that in non-tumor livers (Fig. 1A). It was confirmed by RT-PCR of 9 paired clinical samples (Fig. 1B). Moreover, the analysis of GSE6764 dataset revealed that RPL22L1 had been upregulated in precancerosis and tended to increase with the progression of HCC (Fig. 1C). We also observed RPL22L1 showed an increasing trend along with tumor grades in TCGA-LIHC cohort (Fig. 1D).

To further validate these results, we performed IHC staining in TMA containing 90 paired HCC and peritumoral tissues and found that RPL22L1 was significantly elevated in HCC compared to adjacent tissues (Fig. 1E). In addition, high RPL22L1 expression was positively associated with poorly differentiated grade, large tumor size and high serum AFP level (Table 1). Importantly, elevated RPL22L1 was found to be correlated with shorter overall survival based on TCGA-LIHC dataset and TMA-HCC data (Fig. 1F, G). Univariate and multivariate cox regression analysis displayed that RPL22L1 was an independent prognostic factor for poor overall survival in HCC TMA (Table 2).

**Table 1:** Correlation between RPL22L1 expression and clinicopathological features of HCC in TMA.

Factors		All cases	RPL22L1		*P* value^a^
			Low expression	High expression	
**Gender**					0.116
Female		16	9 (56.3%)	7 (43.7%)	
Male		74	26 (35.1%)	48 (64.9%)	
**Age**					0.631
<52		46	19 (41.3%)	27 (58.7%)	
≥52		44	16 (36.4%)	28 (63.6%)	
**Grade**					**0.045**
I/II		58	27 (46.6%)	31 (53.4%)	
III		32	8 (25.0%)	24 (75.0%)	
**Tumor size (cm)**					**0.008**
≤5.6		54	27 (50.0%)	27 (50.0%)	
>5.6		36	8 (22.2%)	28 (77.8%)	
**Stage**					0.098
I		61	27 (44.3%)	34 (55.7%)	
II		29	8 (27.6%)	21 (72.4%)	
**Recurrence**					0.113
Negative		37	18 (48.6%)	19 (51.4%)	
Positive		53	17 (32.1%)	36 (67.9%)	
**AFP(μg/L)**					**0.009**
<400		57	28 (49.1%)	29 (50.9%)	
≥400		33	7 (21.2%)	26 (78.8%)	

^a^The pearson chi-squared test was used for statistical analysis.

**Table 2:** Univariate and multivariate Cox regression analysis of different prognosis factors in patients with HCC from TMA.

Factors	Cases		Univariate analysis^a^		Multivariate analysis^a^	
			HR (95%CI)	*P* value^b^	HR (95%CI)	*P* value^b^
Total	90					
**RPL22L1 expression**			3.940 (1.607–9.660)	**0.003**	4.352 (1.282–14.775)	**0.018**
Low expression	35					
High expression	55					
**Gender**			1.687 (0.568–5.017)	0.347		
Female	16					
Male	74					
**Age**			1.444 (0.627–3.325)	0.387		
<52	46					
≥52	44					
**Grade**			0.758 (0.319–1.801)	0.530		
I/II	58					
III	32					
**Tumor size (cm)**			2.138 (0.858–5.324)	0.103	0.925 (0.274–3.123)	0.900
≤5.6	54					
＞5.6	36					
**Stage**			3.094 (1.188–8.059)	**0.021**	2.796 (0.780–10.026)	0.115
I	61					
II	29					
**Recurrence**			16.364 (5.686–47.096)	**0.000**	19.316 (5.545–67.292)	**0.000**
Negative	37					
Positive	53					
**AFP(μg/L)**			2.222 (0.912–5.418)	0.079	0.742 (0.215–2.563)	0.637
<400	57					
≥400	33					

^a^Cox regression model.

^b^Log-rank test.

### RPL22L1 promotes proliferation, migration and invasion of HCC cells

**Fig. 2:**
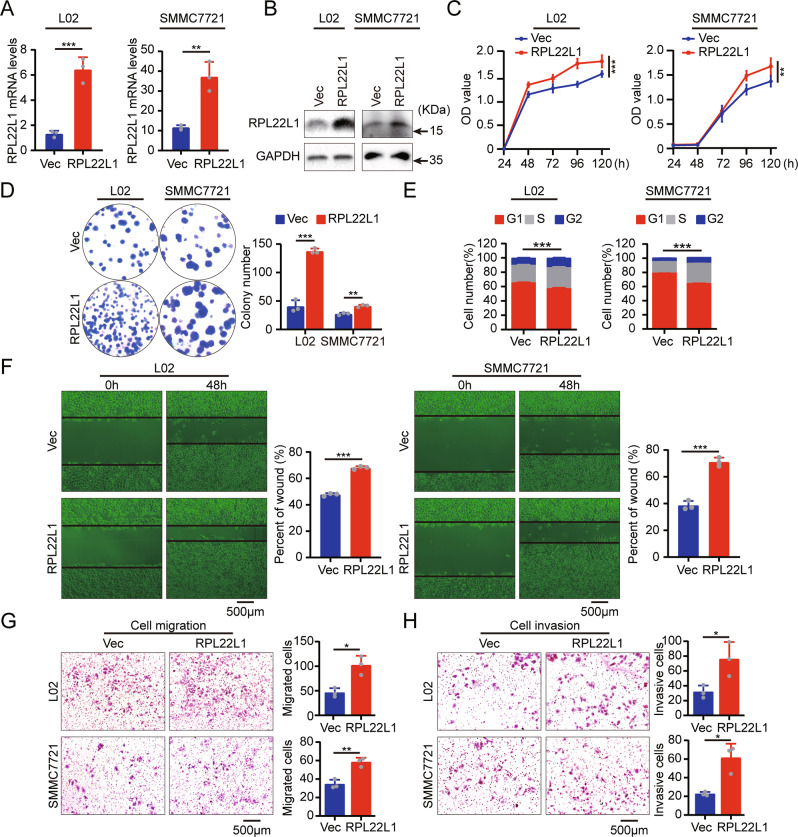
Biological function of RPL22L1 in HCC cells. **A** RT-PCR and **B** western blot verified that two stable RPL22L1-overexpressed cell lines were successfully established. **C** MTS assay showed that RPL22L1 promoted cell viability. **D** Colony formation assay indicated RPL22L1 facilitated cell proliferation. **E** Flow cytometry demonstrated RPL22L1 accelerated cell cycle progression. **F** RPL22L1 promoted cell migration as measured by wound-healing assay. Scale bars = 500 μm. **G** Transwell migration and **H** invasion assays showed RPL22L1 motivated cell migration and invasion. Scale bars = 500 μm. Data are shown as mean ± SD of three independent experiments. Student’s *t*-test. *P < 0.05, **P < 0.01, ***P < 0.001.

To clearly delineate the biological function of RPL22L1 in HCC cells, we established stable RPL22L1 overexpression cell lines based on L02 and SMMC7721 cells (Fig. 2A, B). We found that overexpression of RPL22L1 could significantly promote cell viability and colony formation (Fig. 2C, D). Consistent with this phenotype, cell cycle analysis also showed retarded G1 phase and correspondingly increased S/G2 phase in RPL22L1-overexpressed cells (Fig. 2E, Supplementary Fig. 1A, B). Further, RPL22L1 overexpression significantly promoted migration and invasion of cells as measured by wound-healing and transwell assays, which ruled out the effects of cell proliferation by treatment with the cell proliferation inhibitor mitomycin C (Fig. 2F–H, Supplementary Fig. 1C). These results collectively illustrated the key role of RPL22L1 in promoting HCC cell proliferation, migration and invasion.

### RPL22L1 induces atypical epithelial-to-mesenchymal transition (EMT) of HCC cells by activation of ERK

**Fig. 3:**
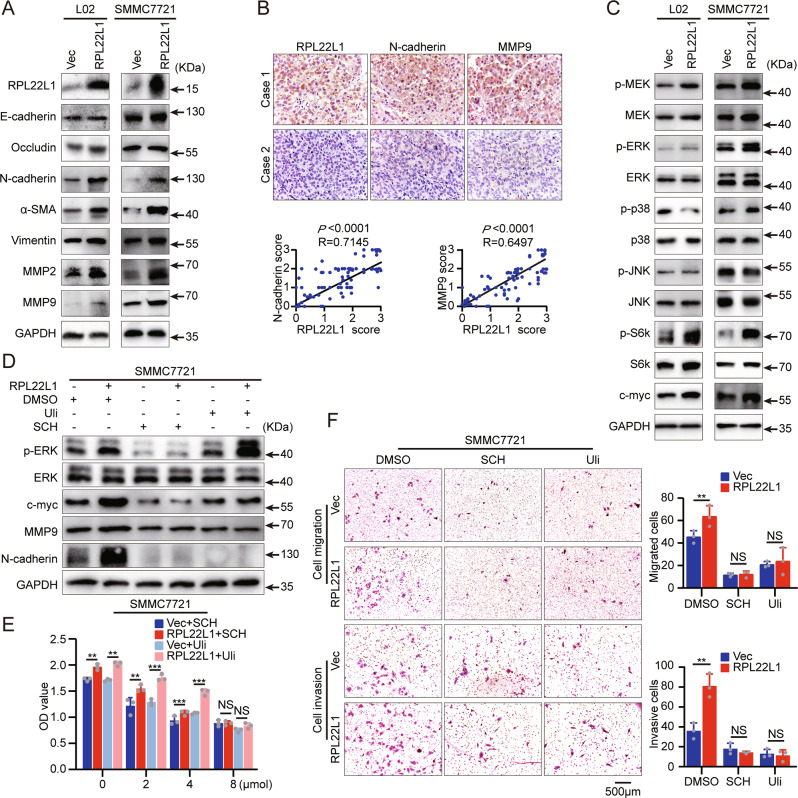
RPL22L1 induces atypical EMT by ERK activation. **A** Western blotting analysis of EMT-associated molecules in RPL22L1-overexpressed L02 and SMMC7721 cells. **B** Representative images of IHC staining in 80 HCC patients. Case 1 was a HCC patient with low RPL22L1 and case 2 was a patient with high RPL22L1. Scale bars = 200 μm. Spearman’s correlation was performed. **C** Western blotting analysis of MAPK pathway-related proteins in RPL22L1-overexpressed L02 and SMMC7721 cells. **D** Western blots showed the effect of ERKi on cells. **E** MTS assays showed dose-dependent effect of ERKi on cell viability. **F** Transwell assays showed the effect of ERKi on cell migration and invasion. Data are shown as mean ± SD of three independent experiments. Student’s t-test. NS non-significant, *P < 0.05, **P < 0.01, ***P < 0.001.

EMT is crucial for the acquisition of malignant properties during cancer progression. We found that RPL22L1 overexpression enhanced the mesenchymal markers N-cadherin, α-SMA and Vimentin expression, although epithelial markers E-cadherin and Occludin had no corresponding changes. Based on the close correlation between matrix metalloproteinase activities and EMT, we also noticed a significant increase in MMP2 and MMP9 levels. (Fig. 3A). In clinical specimens, IHC staining of TMAs containing 80 HCC tissues showed that RPL22L1 was positively correlated with the levels of N-cadherin and MMP9 (Fig. 3B). These results are consistent with RPL22L1 promoting atypical EMT in HCC cells.

On the molecular mechanism, we explored the involvement of MAPKs on RPL22L1-induced EMT. Overexpression of RPL22L1 increased the phosphorylation of MEK, ERK, S6k and the expression of c-myc, while the phosphorylation of p38 and JNK were not affected (Fig. 3C). This suggested the involvement of MEK-ERK pathway in RPL22L1-induced atypical EMT on HCC.

Inhibitors of MEK (U0126, MEKi) and ERK (SCH772984 and Ulixertinib, ERKi) were used to interrogate whether RPL22L1 affected malignant functions of HCC cells by activating MEK-ERK. We found that ERKi completely blocked RPL22L1-promoted expression of c-myc, MMP9 and N-cadherin, as well as cell viability, migration and invasion (Fig. 3D–F). However, MEKi failed to do this (Supplementary Fig. 2). These results suggested that RPL22L1 induced atypical EMT by activating ERK rather than MEK, and finally promoted HCC cells malignant characteristics.

### ERKi potentiates the therapeutic effect of sorafenib in RPL22L1-overexpressed cells

**Fig. 4:**
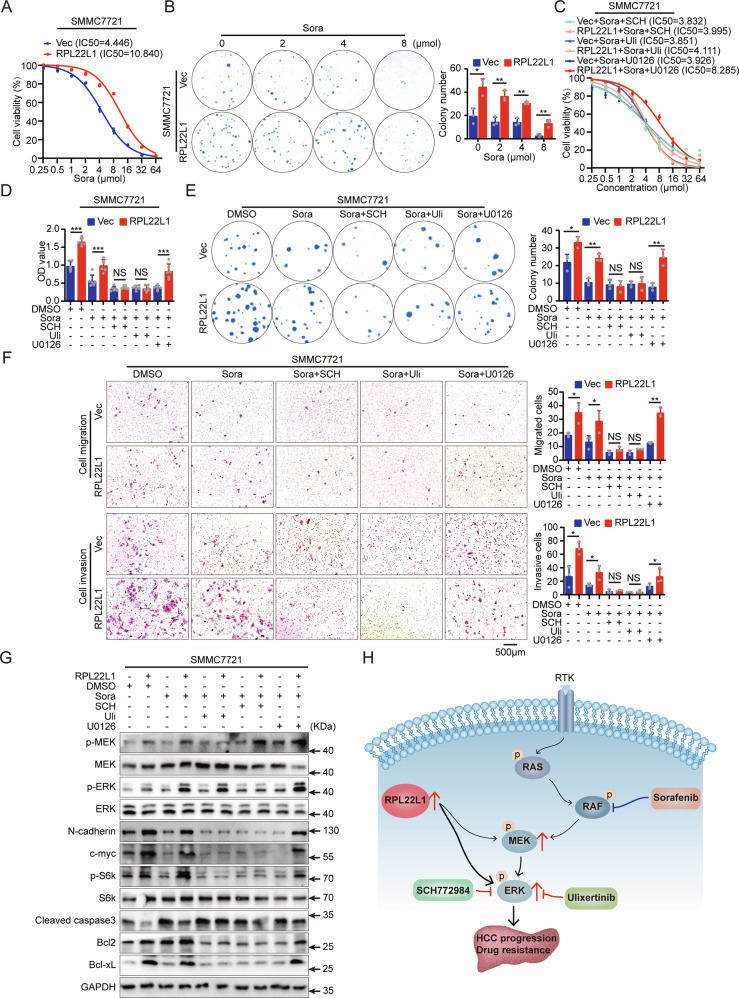
ERKi synergizes with sorafenib in RPL22L1-high cells. **A** MTS assay showed dose-dependent effect of sorafenib on cell viability. IC50 values were calculated. **B** Colony formation of cells treated with different concentrations of sorafenib. Colony numbers were counted. **C** IC50 values of sorafenib combined different ERKi were determined by MTS assays. **D** MTS and **E** colony formation assays showed cell proliferative ability under treatment with sorafenib alone or combination with different ERKi at 8μmol concentration. **F** Transwell assays showed cell migration and invasion capability after treatment with sorafenib alone or combination with different ERKi. Scale bars = 500 μm. Data are shown as mean ± SD of three independent experiments. Student’s t-test. NS non-significant, *P < 0.05, **P < 0.01, ***P < 0.001. **G** Western blots showed the effect of sorafenib alone or combination with different ERKi on cells. **H** Illustration depicted RPL22L1 modulating sorafenib sensitivity via activation of ERK, and the enhancement of sorafenib efficacy by combining with ERKi.

The abnormal activation of MEK-ERK is closely related to the drug resistance of sorafenib, which is the standard first-line treatment for advanced HCC [19, 20]. We further tested the effect of RPL22L1 on sorafenib sensitivity of HCC cells. RPL22L1 overexpression resulted in a poor response to sorafenib, as illustrated by an increased half-inhibitory concentration (IC50) and clonogenic growth (Fig. 4A, B).

We next analyzed the effect of MEKi (U0126) or ERKi (SCH772984 and Ulixertinib) in combination with sorafenib. The combination with ERKi significantly reduced the IC50 of sorafenib and showed a synergy in inhibiting the proliferation, migration and invasion of RPL22L1-overexpressed cells, but the effect on control cells was not so obvious. However, U0126 and sorafenib had little synergy in RPL22L1-overexpressed cells (Fig. 4C–F).

Sorafenib didn’t block the levels of p-MEK, p-ERK, p-S6k, c-myc and N-cadherin in RPL22L1-overexpressed cells. Besides, overexpression of RPL22L1 led to a decrease of Cleaved caspase3, accompanied by the increase of Bcl2 and Bcl-xL in sorafenib treatment. However, the combination of sorafenib with ERKi could effectively reverse these molecular changes, although the level of p-MEK was still increased (Fig. 4G). To summarize, these results clearly delineated RPL22L1 restrict sorafenib response by activating ERK, and suggested that ERKi combined with sorafenib may be an effective treatment regimen for HCC patients with high expression of RPL22L1 (Fig. 4H).

## Discussion

High heterogeneity of HCC can lead to different responses to therapy, and thus highlight the importance of precision medicine approaches. Herein this study, we determined the functional role of RPL22L1 and its potential therapeutic relevance in HCC.

In this study, we found that RPL22L1 was not only highly expressed in HCC tissues, but also tended to increase with HCC progression by using public database and analysis of clinical samples. Further, clinical data showed that upregulation of RPL22L1 in HCC patients was strongly correlated with tumor differentiation, tumor size and serum AFP levels. At present, AFP is the only biomarker with proven diagnostic and prognostic value in advanced HCC and associated with sorafenib resistance [21, 22]. Our survival analysis revealed poor prognosis for HCC patients with high RPL22L1 expression. Furthermore, univariate and multivariate Cox regression analysis indicated RPL22L1 was an independent predictor of poor prognosis in HCC patients, supporting that RPL22L1 could be a potential target in HCC diagnosis and treatment.

Subsequently, we confirmed RPL22L1 could substantially promoted HCC cells proliferation, migration, invasion and atypical EMT. Recent studies have shown that EMT in tumors may undergo a process of gradually co-expressing epithelial and mesenchymal markers, which suggests tumor cells undergo atypical EMT [23]. Such EMT heterogeneity was demonstrated to contribute to increased metastatic potential and drug resistance [24, 25]. In our study, RPL22L1 overexpression induced atypical EMT process in which E-cadherin and Occludin didn’t altered accordingly. Clinical relevance analysis also demonstrated the significant correlation between RPL22L1 and atypical EMT. Therefore, HCC cells with high RPL22L1 expression probably undergo atypical EMT to achieve higher malignant behavior.

The MAPK pathway is related to tumor metastasis, atypical EMT, and drug resistance [26**–**29]. Three major MAPK subfamilies in mammals have been identified: JNK, p38 and ERK1/2. JNK and p38 are closely related to stress and apoptosis of cells, while ERK1/2 plays a vital role in several steps of tumorigenesis including cell proliferation, migration and invasion [30, 31]. In this study, ERK1/2 and its upstream MEK1/2 were activated, owing to increased expression of RPL22L1, while the phosphorylations of p38 and JNK were unaffected. Moreover, RPL22L1-caused adverse consequence were completely reversed by ERKi (SCH772984 and Ulixertinib). This indicates that RPL22L1 may promote atypical EMT by activating ERK and further promote the malignant progression of HCC. Further work is needed on the mechanisms by which RPL22L1 activates ERK.

Abnormal activation of ERK via multiple mechanisms is an important cause of sorafenib resistance in advanced HCC [32, 33]. We here demonstrated that RPL22L1 limited therapeutic activity of sorafenib by activating ERK. Combination therapies can help improve response to approved drugs and fight therapy failure. ERKi, SCH772984 and Ulixertinib, are undergoing clinical evaluation and hold a promising application for cancer treatment. We confirmed ERKi (SCH772984 and Ulixertinib) achieved synergistic efficacy with sorafenib in RPL22L1-overexpressed cells. The two ERKi display distinct mechanism of action, with Ulixertinib (Catalytic ERKi) solely preventing ERK1/2 catalytic activity while SCH772984 (Dual mechanism ERKi) extra blocking p-ERK1/2 at T-E-Y motif by MEK1/2 [34]. This probably explained the discrepancy in p-ERK level between the two ERKi. However, U0126 unfortunately remained ineffectual due to no significant effect on ERK activity. Recent studies have also shown that U0126 resistance correlated with MEK hyperphosphorylation and the lack of prolonged ERK suppression [35].

HCC cells with high RPL22L1 expression exhibit poor therapeutic efficacy of sorafenib and require combination with ERKi therapy. Precision medicine is needed to maximize drug benefit and minimize harm. Our data demonstrate that RPL22L1 overexpression promotes HCC progression and sorafenib resistance by activation of ERK. Combining sorafenib and ERKi shows a synergistic effect in RPL22L1-high HCC cells. Hence, our findings lay a theoretical foundation for RPL22L1 to become a potential emerging precision medicine marker for HCC diagnosis and treatment.

## Materials and methods

### Ethics approval and consent to participate

This work was approved by the Ethics Committee of Harbin Medical University (No. HMUIRB20150023, Harbin, China). Informed consents were obtained from all patients.

### Data acquisition

RPL22L1 mRNA level was analyzed based on TCGA (LIHC dataset, http://www.tcga-data.nci.nih.gov) and NCBI GEO databases (GSE64041, GSE45436, GSE112790, GSE121248, GSE84402, GSE17548, GSE62232, GSE136247, GSE59259, GSE25097, GSE36376, GSE46408, GSE98383, GSE6764). Survival analysis of RPL22L1 in TCGA-LIHC dataset was performed using GEPIA database (http://gepia.cancer-pku.cn/).

### Clinical specimen and tissue microarrays (TMA)

Eighteen clinical specimens including HCC (*n* = 9) and their peritumor (*n* = 9) were obtained upon surgical resection from the Second Affiliated Hospital of Harbin Medical University (Harbin, China). Human HCC TMAs HLivH180Su07HE (90 paired HCC and peritumor tissues) and HLivH160CS01 (80 paired HCC and adjacent non-neoplastic tissues) were obtained from Outdo Biotech CO. Ltd. (Shanghai, China).

### Cell lines and reagents

HCC cell line SMMC7721 (CBP60210) and normal hepatocyte cell line L02 (CBP60224) were purchased from the Cell Bank of the Chinese Academy of Sciences (Shanghai, China). Cells were cultured in the media in the prescribed manner, and were certified by STR in the Micro-read Genetics Company (Beijing, China). Mitomycin C (HY-13316), Sorafenib (HY-10201), U0126 (HY-12031A), Ulixertinib (HY-15816) and SCH772984 (HY-50846) were purchased from MedChemExpress (Monmouth Junction, NJ, USA).

### Real-time polymerase chain reaction (RT-PCR)

The total RNA was isolated using Trizol Reagent (Invitrogen, Carlsbad, CA) and then transcribed into cDNA with Transcriptor First Stand cDNA System Kit (Roche, Basel, Switzerland). The quantification of cDNA was conducted using LightCycler ^®^ 480 SYBR Green I Master (Roche). The cDNA levels were normalized to β-actin. The sequences of primers used were listed as follows: RPL22L1-F, 5′-AGAAGGTTAAAGTCAATGG-3′, RPL22L1-R: 5′-ATCACGAAGATTGTTCTTC-3′; β-actin -F: 5-CATGTACGTTGCTATCCAGGC-3′, β-actin -R: 5′-CTCCTTAATGTCACGCACGAT-3′.

### Transfection

Lentivirals expressing RPL22L1 or vector were purchased from HANBio (Shanghai, China) and transfected into cells using polybrene (HANBio, Shanghai, China) according to the manufacturer’s instructions.

### Immunohistochemistry (IHC)

IHC staining was performed with PowerVision™ Two-Step Histostaining Reagent (Zhongshan Golden Bridge, Beijing, China) according to the manufacturer’s instructions. The slides were incubated with rabbit polyclonal antibody (Supplementary Table 1) overnight at 4 °C. Counterstaining was performed with hematoxylin. Negative controls were performed with normal rabbit IgG instead of primary antibody. Immunoreactivity intensity was scored as 0, 1, 2, or 3 based on a consensus of three researchers.

### Western blotting

Protein extracts were obtained from cells with RIPA buffer (Thermo Fisher Scientific, Waltham, MA, USA), then loaded onto 10–12% SDS-PAGE and transferred to PVDF membranes (Millipore, Billerica, MA, USA). After blocking with 5% BSA, proteins were incubated with primary antibodies (Supplementary Table 1) overnight at 4 °C and anti-mouse/rabbit secondary antibody (Cell signaling technology, Boston, USA) for 2 h at room temperature. The bands were scanned using the ChemiDoc TM MP Imaging System (BIO-RAD).

### Colony formation assay

Cells were plated into 6-well plates (L02: 1000 cells/well; SMMC7721: 700 cells/well) and subsequently treated with drugs for approximately 2 weeks. The supernatant was renewed every 3 days with medium containing fresh drugs until visible colonies formed. Colonies were fixed with 4% paraformaldehyde for 15 min and then dyed with 0.5% crystal violet for 20 min.

### Wound healing assay

The cell monolayer was scratched with 10 μl pipette tip in a 6-well plate. The gap width was recorded at 0 h, 24 h and 48 h with light microscope (Leica Microsystems). The rate of migration was analyzed as the percentage of wound healing.

### Cell migration and invasion assay

Transwell migration and invasion assays were conducted using corning chambers (Corning, MA, USA) with or without matrigel. A total of 5 × 10^4^ cells in media with 2% fetal bovine serum (FBS) were placed in upper chambers, while media containing 20% FBS was added into the lower chambers. After incubating for 48 h at 37 °C, the migrated or invaded cells were fixed with 4% paraformaldehyde, and stained with hematoxylin and eosin (H&E). Cells were counted and photographed.

### Cell proliferation assay

Cell proliferation assays were performed using the MTS kits (Promega, Madison, Wisconsin, USA). Cell suspensions (1000 cells/well) were inoculated in a 96-well plate. After culturing for 24 h, the old mediums were discarded. Mixtures of MTS and medium were added into each well (Reagent: media = 1:4). Optical density (OD) values at 490 nm wavelength were measured after 2 h cultured.

### Flow cytometry

Cells were harvested and washed in PBS, and then fixed in ice-cold 70% ethanol at 20 °C for 2 h. After centrifugation, cells were washed with PBS, and treated with 100 μg/ml RNase A (Thermo Fisher Scientific, EN0531) and 50 μg/ml propidium iodide (Sigma-Aldrich, P4864) for 30 min at 37 °C in the dark. Cell cycle status was evaluated on an Accuri C6 Plus flow cytometer (BD Biosciences, SanJose, CA, USA), and data were analyzed using Modfit (Verity, Topsham, ME, USA) software programs.

### Statistical analysis

The SPSS 23.0 statistical package (SPSS Inc. Chicago, IL, USA) was used for calculation. Wilcoxon signed-rank test was performed for RPL22L1 expression difference in TMA. χ^2^ test was used to determine the correlation between RPL22L1 and clinic-pathological factors. Survival curves were plotted by the Kaplan-Meier method and analyzed by log-rank test. Cox proportional hazards regression model was performed for univariate and multivariate survival analysis. Kolmogorov Smirnov test was used to detect the normal distribution of quantitative data, ANOVA was used to detect the homogeneity of variance before two-tailed Student’s t-test performed to detect the difference between groups. All data are shown as mean ± SD. *P* < 0.05 for the difference was considered statistically significant.

## Supplementary information

Supplementary Figure and Table legends

author contribution statement

Supplementary Figure 1

Supplementary Figure 2

Supplementary Table 1

Original western blots

Original western blots

Original western blots

Original western blots

Ethical review of this study

## Data Availability

Data are available on request to the corresponding author.
