# Supplementary material for: Ribosomal protein L22-like1 (RPL22L1) mediates sorafenib sensitivity via ERK in hepatocellular carcinoma
Source: Cell Death Discov. 2022 Aug 17;8:365. doi: 10.1038/s41420-022-01153-8 (PMC9381560; doi:10.1038/s41420-022-01153-8)
Supplement: Supplementary file 1 — Supplementary Figure and Table legends [file 41420_2022_1153_MOESM1_ESM.docx]

**Supplementary Figure and Table legends**

**Supplementary Figure 1. Supplementary data for the effect of RPL22L1 on cell proliferation.**

(A-B) Representative flow cytometry analysis of L02 (A) and SMMC7721 (B) cells. (C) MTS assay showed the changes of cell proliferation at 48h after treatment with 40ug/ml Mitomycin C for 5min. Data are shown as mean ± SD. Student’s t-test. NS non-significant.

**Supplementary Figure 2. Supplementary data for the effect of MEKi on cells.**

(A) Western blots showed the effect of MEKi on cells. (B) MTS assays showed dose-dependent effect of MEKi on cell viability. (C) Transwell assays showed the effect of MEKi on cell migration and invasion. Data are shown as mean ± SD of three independent experiments. Student’s t-test. NS non-significant, * *P*<0.05, ** *P*<0.01, *** *P*<0.001.

**Supplementary Table 1. Primary antibodies.**
