# Supplementary material for: Ribosomal protein L22-like1 (RPL22L1) mediates sorafenib sensitivity via ERK in hepatocellular carcinoma
Source: Cell Death Discov. 2022 Aug 17;8:365. doi: 10.1038/s41420-022-01153-8 (PMC9381560; doi:10.1038/s41420-022-01153-8)
Supplement: Supplementary file 2 — author contribution statement [file 41420_2022_1153_MOESM2_ESM.pdf]

DECLARATION OF CONTRIBUTIONS TO ARTICLE

ADMC

Manuscript Number: CDDISCOVERY-22-4467R

Journal Name: *Cell Death Discovery* (the 'Journal')

Proposed Title of the Contribution: Ribosomal protein L22-like1 (RPL22L1) mediates sorafenib sensitivity via ERK in hepatoce llular carcinoma (the 'Contribution)

Author(s): Dongmei Zhang, Yunzhen Zhou, Yanan Ma, Ping Jiang, Hongchao Lv, Sijia Liu, Yu Mu, Chong Zhou, Shan Xiao, Guohua Ji, Peng Liu, Ning Zhang, Donglin Sun, Haiming Sun, Nan Wu and Yan Jin (the 'Authors')

For all *CDDis*covery articles, each person named as an author in the published version must be able to show he or she has contributed substantially to the article.

Authorship credit should be based on 1) substantial contributions to conception and design, acquisition of data, or analysis and interpretation of data; 2) drafting the article or revising it critically for important intellectual content; and 3) final approval of the version to be published. Authors should meet conditions 1, 2 and 3.

Any person who cannot be shown to have made a substantial contribution to the article cannot be listed as an author in the final version. The name of any person who is deemed to have made a minor contribution can, however, appear in the Acknowledgments section of the article.

Please complete the table below to indicate the contributions of all named authors to the manuscript.

Author Full Name:	Specification of Contribution to the Manuscript:
Dongmei Zhang	Performed the experiments, Wrote the paper.
Yunzhen Zhou	Performed partial experiments.
Yanan Ma	Participated in functional assays.
Ping Jiang	Participated in functional assays.
Hongchao Lv	Analyzed data.
Sijia Liu	Participated in functional assays.
Yu Mu	Involved in mechanism study.
Chong Zhou	Took charge of bioinformatics analyses.
Shan Xiao	Involved in mechanism study.
Guohua Ji	Provided technique supports.
Peng Liu	Provided technique supports.
Nan Wu	Embellished the manuscript, responsible for funding.
Yan Jin	Embellished the manuscript, responsible for funding.

Please complete the table below to indicate the contributions of all named authors to the figures.

Figure 1:

Dongmei Zhang, Nan Wu, Hongchao Lv and Chong Zhou analyzed data. Dongmei zhang and Sijia Liu performed immunohistochemistry.

Figure 2:

Dongmei Zhang and Yunzhen Zhou performed western blot analysis. Dongmei Zhang, Ping Jiang, Yanan Ma and Shan Xiao performed MTS, colony formation, wound healing and transwell assays.Donglin Sun, Haiming Sun, Peng Liu and Guohua Ji provided guidance.

Figure 3:

Dongmei Zhang and Yu Mu performed western blot. Dongmei Zhang finished immunohistochemistry.

Figure 4:

Dongmei Zhang and Yunzhen Zhou carried out western blot and colony formation assays. Yunzhen Zhou performed MTS and transwell assays. Ning Zhang involved in colony formation assays.

Figure 5:

Figure 6:

Signed for and on behalf of the Author(s):	Print Name:	Date:
Yan Jin	Yan Jin	2022-07-23
