# Supplementary figures and images for: Ribosomal protein L22-like1 (RPL22L1) mediates sorafenib sensitivity via ERK in hepatocellular carcinoma
Source: Cell Death Discov. 2022 Aug 17;8:365. doi: 10.1038/s41420-022-01153-8 (PMC9381560; doi:10.1038/s41420-022-01153-8)

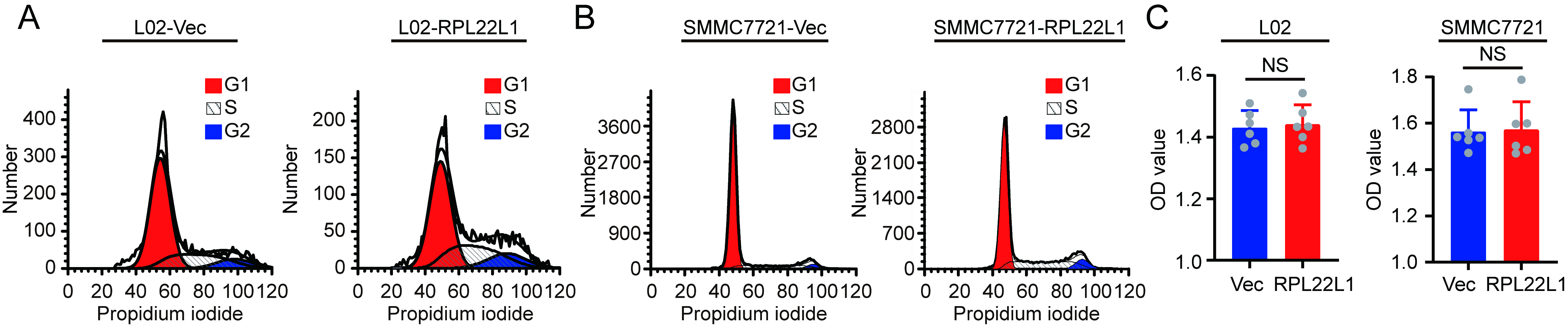

Supplement: Supplementary file 3 — Supplementary Figure 1 [file 41420_2022_1153_MOESM3_ESM.tif]

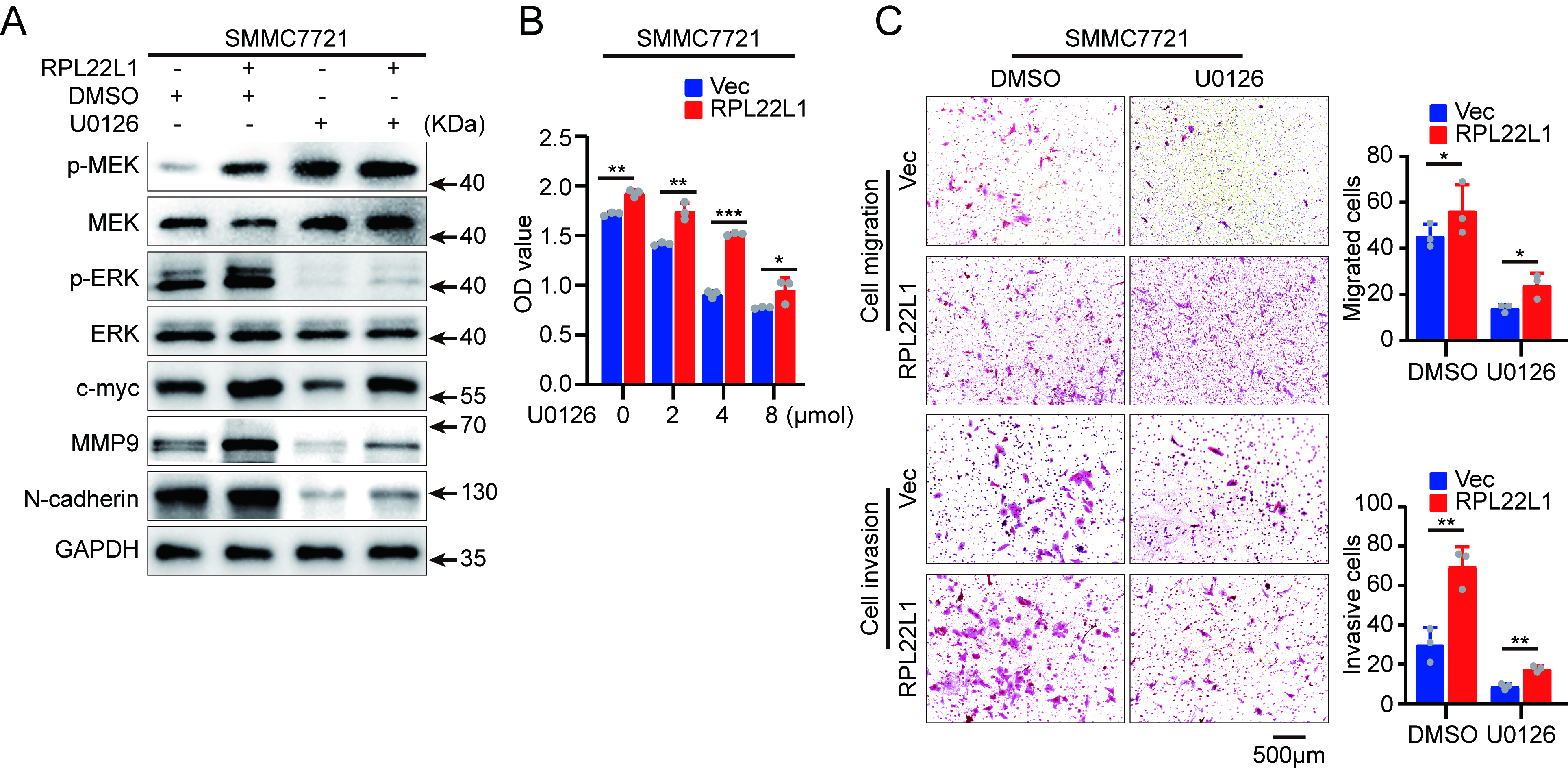

Supplement: Supplementary file 4 — Supplementary Figure 2 [file 41420_2022_1153_MOESM4_ESM.tif]

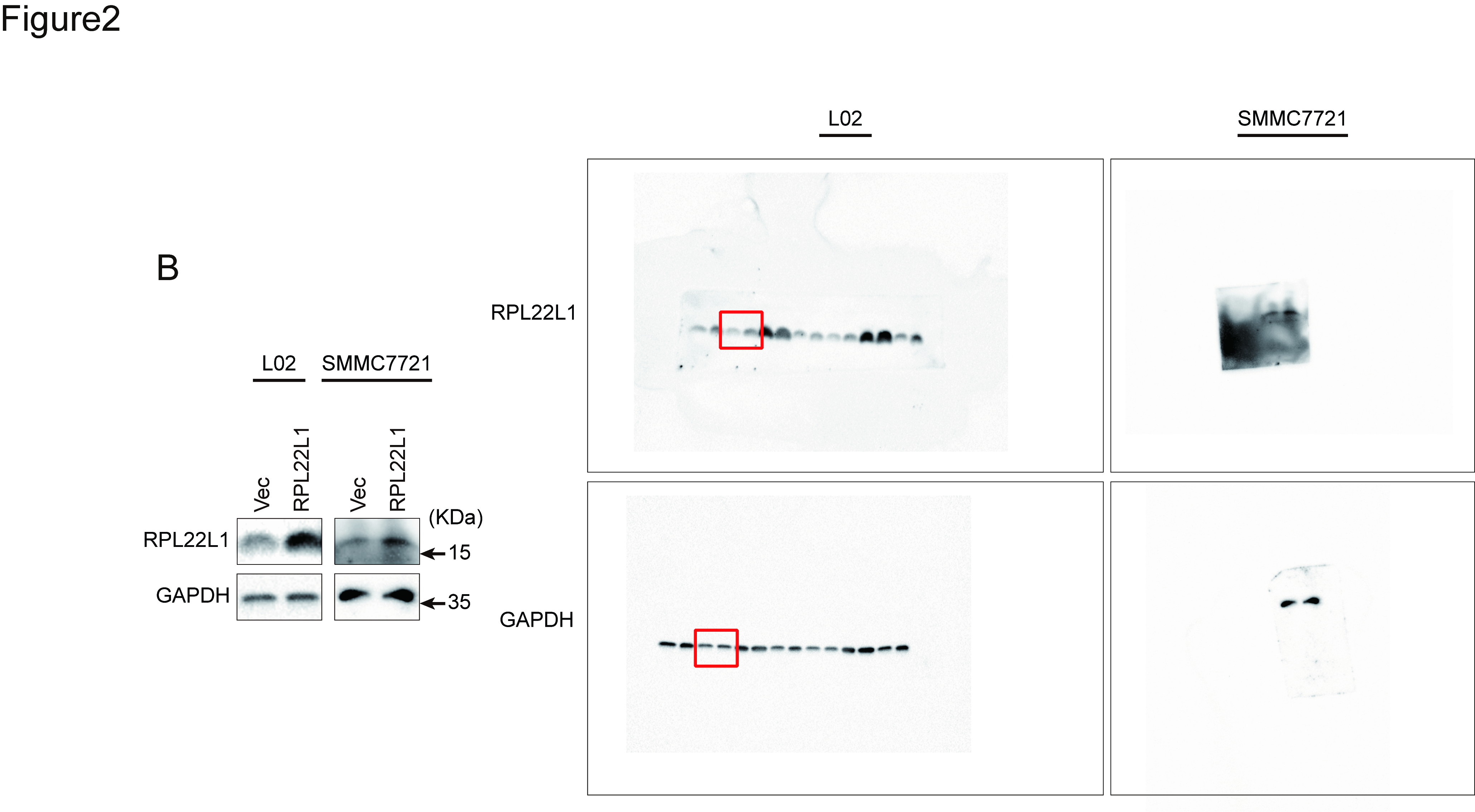

Supplement: Supplementary file 6 — Original western blots [file 41420_2022_1153_MOESM6_ESM.tif]

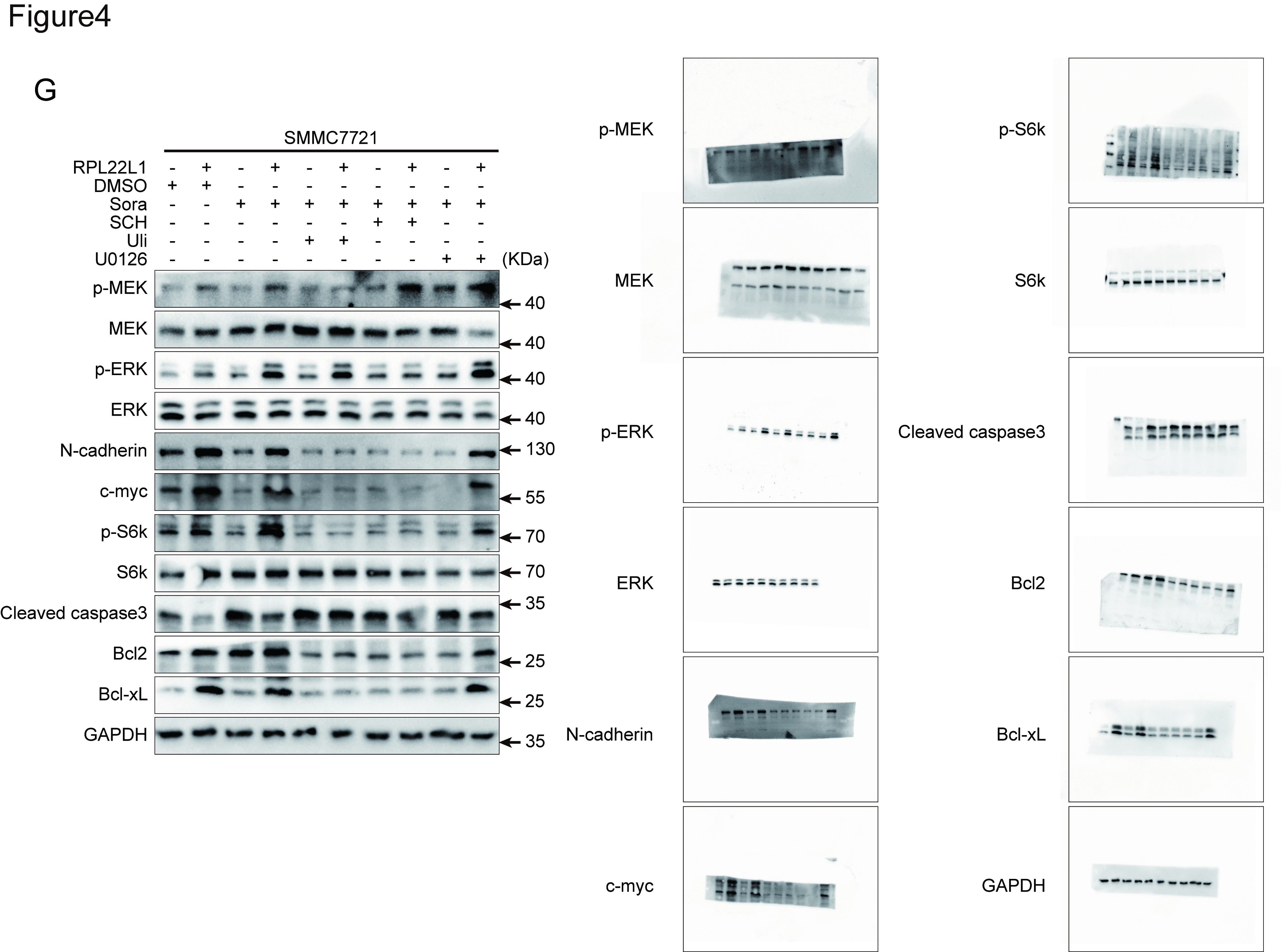

Supplement: Supplementary file 8 — Original western blots [file 41420_2022_1153_MOESM8_ESM.tif]

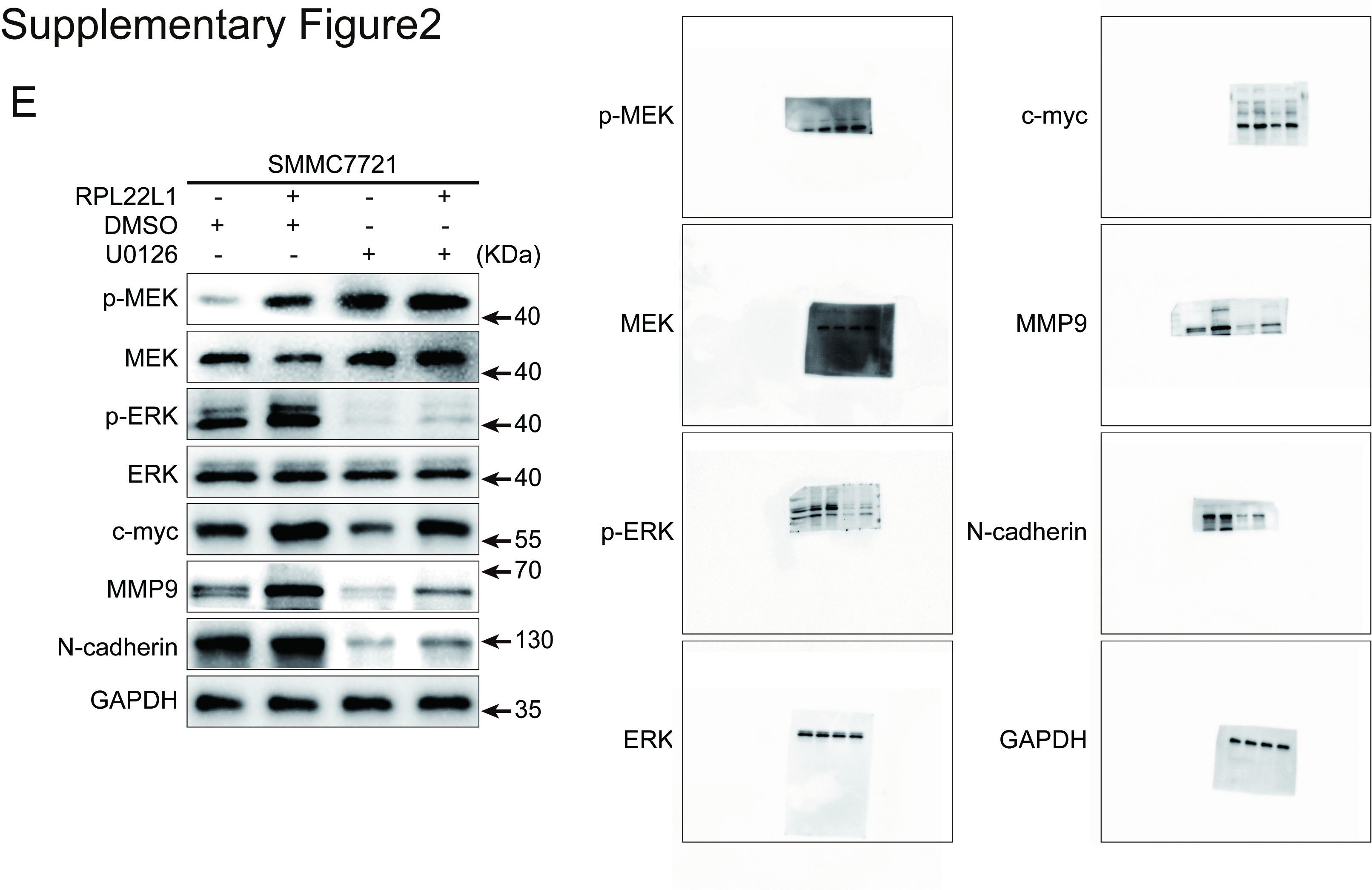

Supplement: Supplementary file 9 — Original western blots [file 41420_2022_1153_MOESM9_ESM.tif]
