# Supplementary material for: Ribosomal protein L22-like1 (RPL22L1) mediates sorafenib sensitivity via ERK in hepatocellular carcinoma
Source: Cell Death Discov. 2022 Aug 17;8:365. doi: 10.1038/s41420-022-01153-8 (PMC9381560; doi:10.1038/s41420-022-01153-8)
Supplement: Supplementary file 10 — Ethical review of this study [file 41420_2022_1153_MOESM10_ESM.pdf]

Harbin Medical University

Institutional Research Board Report

No.: HMUIRB20150023

Laboratory of Genetics of Harbin Medical University intends to carry out the research of "Study on the function of double minute amplified gene *RPL22L1* and its application in molecular diagnosis of tumor". The program is based on the "human samples and animal" as the objects of study. Ethical board approval from the Human Ethics Review Board, Harbin Medical University is on the process.

1. Project Information

Research project name: Study on the function of double minute amplified gene *RPL22L1* and its application in molecular diagnosis of tumor

Undertaking Department: Laboratory of Genetics of Harbin Medical University

Project Director: Yan Jin **Title:** Prof.

Beginning and ending date of the research: 2013/01-2015/12

2. Main Research Contents

The relationship between *RPL22L1* and cancer is unknown. To explore the effect of *RPL22L1* on the development of tumor, immunohistochemical analysis of clinical specimens was performed. Clinical tissue microarrays were purchased from US BIOMAX （Rockville, MD, USA), and XinChao (HOva-Can90PT-01; Shanghai, China). Other samples were collected from archives of paraffin-embedded tissues obtained at the Department of pathology, The Third Affiliated Hospital of Harbin Medical University (Tumor Hospital of Harbin Medical University) (Harbin, Heilongjiang, China). Clinical frozen tissues were collected by The Third Affiliated Hospital of Harbin Medical University, qRT-PCR and western blot were used to detected expression of RPL22L1 or other related proteins in frozen tissues.The human ovarian cancer cell line UACC-1598, SKOV3 and colorectal cancer cell line LoVo, SW620 were purchased from the Type Culture Collection of the Chinese Academy of Sciences (Shanghai, China), human colorectal cancer cell line SW480 and SK-CO-1 were purchased from American Type Culture Collection (ATCC, Manassas, VA, USA).Effect of *RPL22L1* on the xenograft tumor development in nude mice. Tumor cells were subcutaneously / tail vein / intraperitoneal / spleen injection in nude mice, detected the effect of *RPL22L1* on the xenograft tumorigenesis and development.Effect of *RPL22L1* on the metastasis ability of xenograft tumor in nude mice. The xenograft tumors were paraffin embedded and the slices were stained by hematoxylin and eosin (HE) or immunohistochemistry to detect the expression of RPL22L1 or other metastasis related proteins. The expression of *RPL22L1* or other related genes were also detected by qRT-PCT or western blot in fresh tissues.

3. Review evaluation opinions

Safety and fairness principle has been fully considered in the experiments plans. All of the volunteers gave written informed consent, and the content of the research have no harm or risk. Before the research was conducted, ethical board approval from the Harbin Medical University was obtained. No conflict of interest exists in this study. Based on the deliberation of the Medical Ethics Review Committee of Harbin Medical University, this research must be carried out with the experimental animals, and it can't be done with nonliving methods such as computer simulation replacing animals, or with the lower animals replacing the higher animals. The breeds, quality levels and specifications of the animals used in the experiment are all qualified. The researchers have already optimized the experimental scheme and treated the animals kindly by improving experiment method and adjusting the observation index of the experiment to ensure the implementation of the animal welfare measures.

4. Conclusion

The rights and interests of volunteer subjects have been adequately protected in the study, and there is no potential risk to the volunteers. The rights and interests of experimental animals are completely protected, so the tested animals don't have potential risks. It is agreed that the research project goes on as planned.

Institutional Research Board of Harbin Medical University

10/12/2015
